# Dysphagia Lusoria: A Rare Case of Dysphagia

**DOI:** 10.7759/cureus.71235

**Published:** 2024-10-10

**Authors:** Ana Franco, Ana Mordomo, João Ribeiro

**Affiliations:** 1 Family Medicine, Unidade de Saúde Familiar (USF) Arco-íris, Unidade de Saúde Familiar (ULS) Amadora/Sintra, Lisboa, PRT; 2 General Surgery, Unidade de Saúde Familiar (ULS) Amadora/Sintra, Lisboa, PRT

**Keywords:** aberrant subclavian artery, congenital vascular anomaly, ct angiography, dysphagia, dysphagia lusoria

## Abstract

Dysphagia is a condition whose prevalence increases with age and can have multiple causes. The differential diagnosis of dysphagia is crucial for its management and therapeutic guidance. The existence of an aberrant right subclavian artery can be a cause of dysphagia in adults.

We describe a case of a 68-year-old man who consulted his family doctor in primary healthcare due to recurrent complaints of dysphagia for liquids, which had been evolving for several years. Following the investigation with complementary diagnostic tests, an aberrant right subclavian artery was identified and named as the probable cause of the symptoms presented. Lusory dysphagia is an anomaly in the swallowing process caused by extrinsic esophageal compression due to an aberrant subclavian artery.

This case report aims to alert to the possibility of the existence of a congenital malformation as a cause of dysphagia in adults, emphasizing the importance of considering this diagnosis when making the differential diagnosis of this condition.

## Introduction

The term dysphagia refers to an anomaly in the swallowing process, which can affect around 7% of the population. This prevalence increases with age, and it is estimated to be 30-40% in the elderly population [1].

There are multiple causes of dysphagia, including anatomical abnormalities in the upper gastrointestinal tract and functional conditions of the muscular and/or nervous systems. Dysphagia can be divided into oropharyngeal and esophageal dysphagia, according to its pathophysiology. Among oropharyngeal dysphagia, 80% of cases are caused by diseases of the neurologic system, and another important cause, although less frequent, is head and neck neoplasms. Regarding esophageal dysphagia, more than 85% of cases are caused by gastrointestinal diseases, whether structural (esophageal diverticulum) or functional (esophageal reflux disease) [1,2]. Precise identification of the cause of dysphagia is crucial for therapeutic guidance.

## Case presentation

A 68-year-old man consulted his family doctor due to frequent complaints of dysphagia for liquids, which had been evolving for several years with recent worsening. He also reported a chronic, productive cough, worsening in the morning. He denied other symptoms such as weakness, gait or balance disorders, tremors, speech difficulties, breathing difficulty, sialorrhea, weight loss, vomiting, anorexia, heartburn, or other accompanying symptoms.

The patient's past medical history highlights gastroesophageal reflux disease, hypertension, hypertriglyceridemia, overweight, and psoriasis. He had ethanolic habits with regular consumption of 350 g/week and had been a smoker until 20 years ago, with a smoking history of 30 pack-years. He denied drug use. The patient was a retired taxi driver, having had significant environmental exposure to smoke and pollution for around four decades. The clinical examination revealed no appreciable changes.

**Figure 1:**
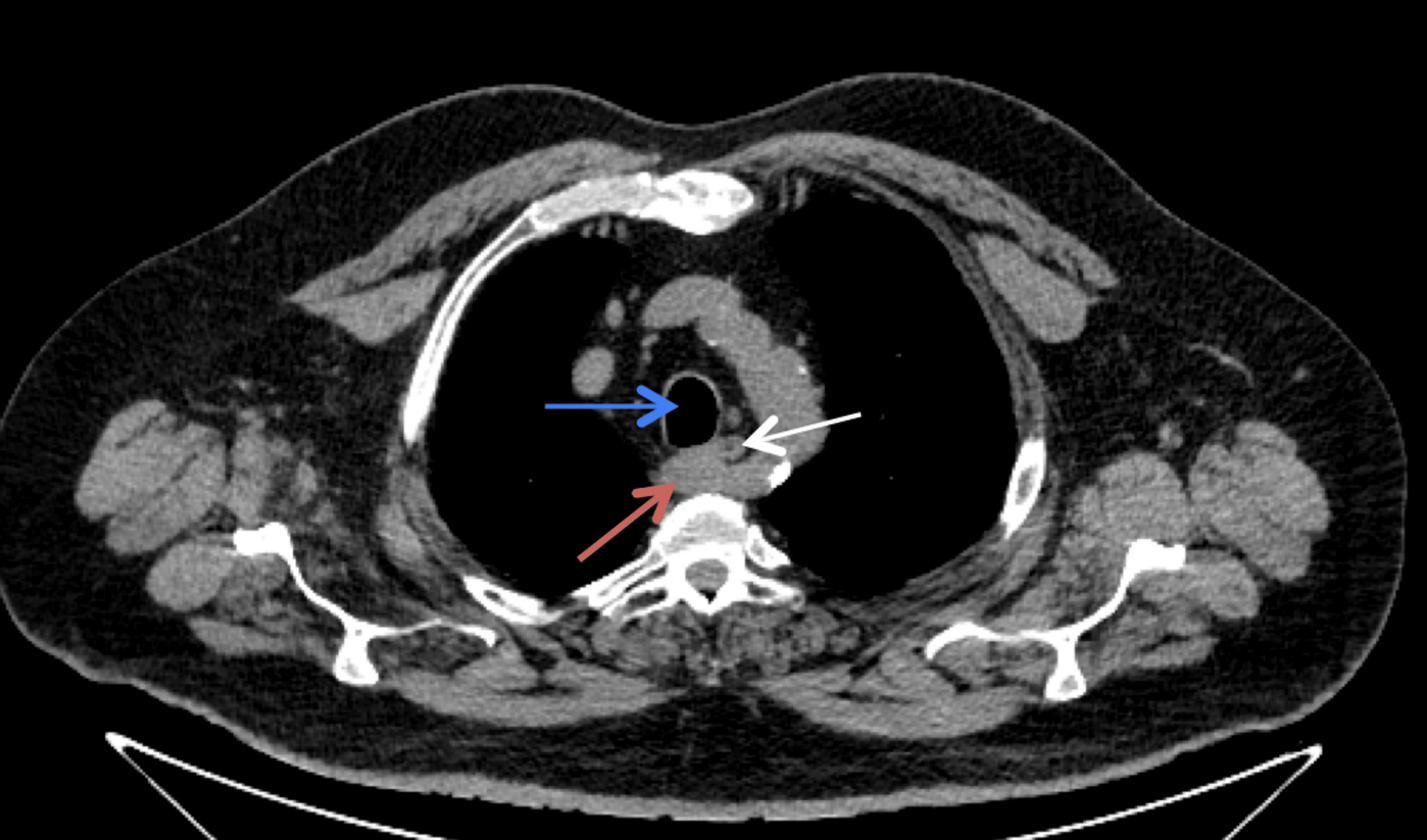
Thoracic CT scan (axial view) showing the aberrant subclavian artery (red arrow), thoracic esophagus (white arrow), and trachea (blue arrow) CT: computed tomography

**Figure 2:**
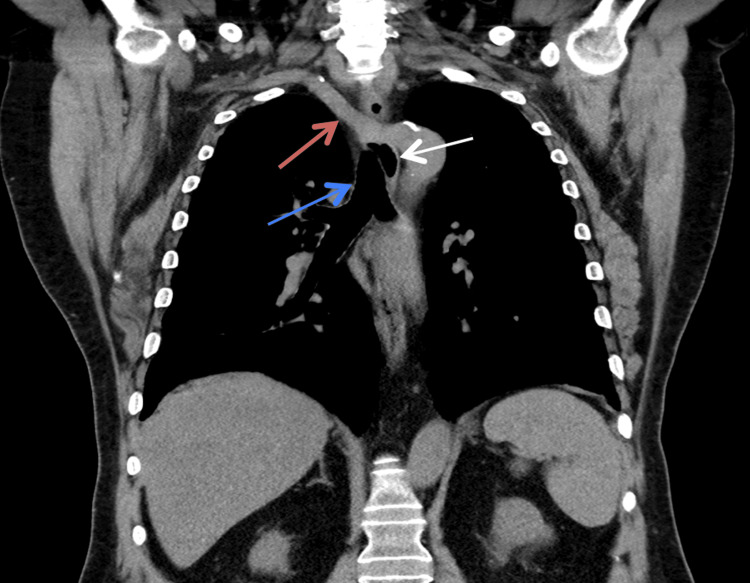
Thoracic CT scan (coronal view) showing the aberrant subclavian artery (red arrow), thoracic esophagus (white arrow), and trachea (blue arrow) CT: computed tomography

**Figure 3:**
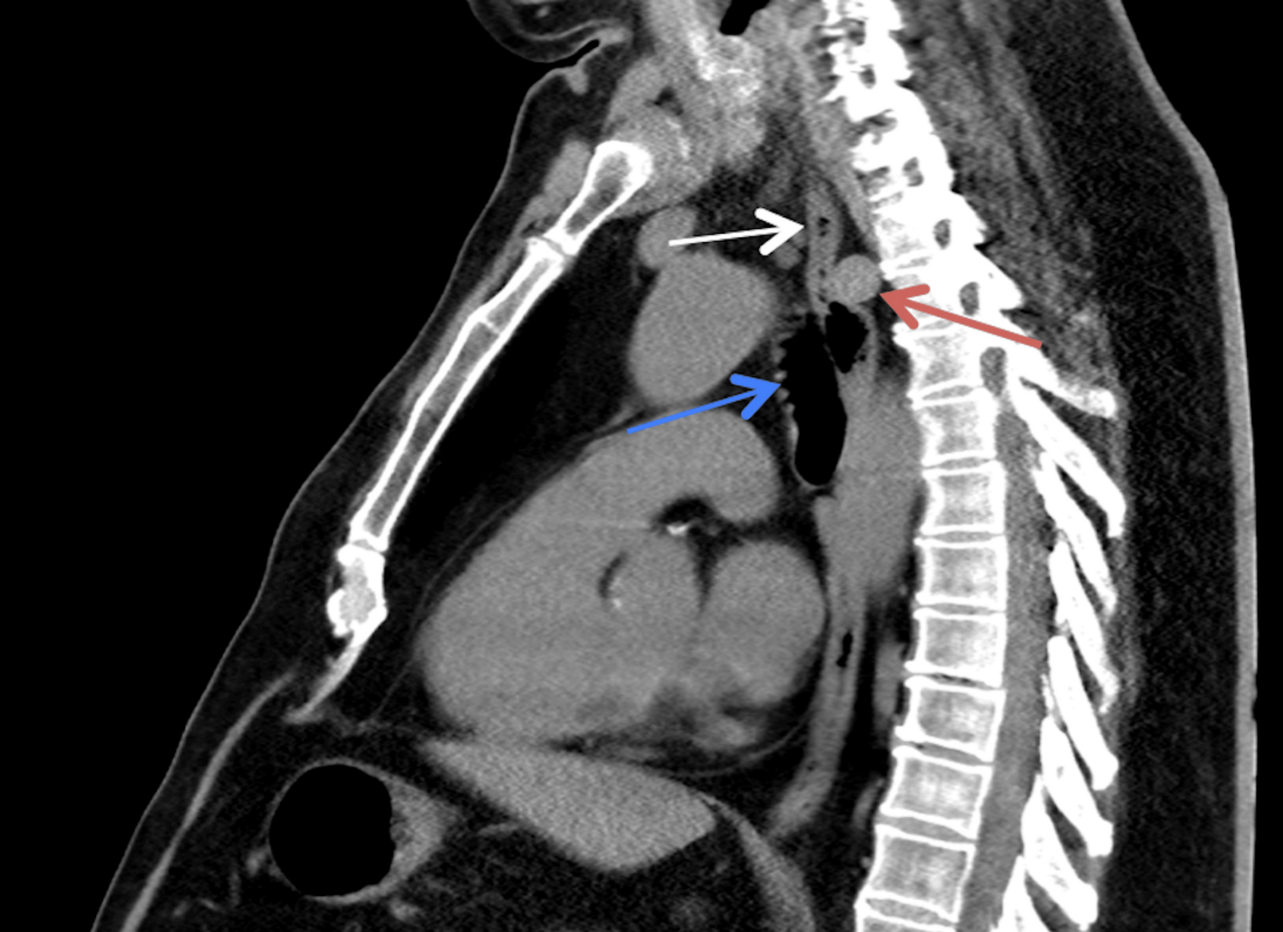
Thoracic CT scan (sagittal view) showing the aberrant subclavian artery (red arrow), thoracic esophagus (white arrow), and trachea (blue arrow) CT: computed tomography

In this context, an esophagogastroduodenoscopy was performed, which revealed a small sliding hiatal hernia and four chronic erosions of the antrum whose anatomical pathology was compatible with focal glandular hyperplasia, with edema of the lamina propria. A chest X-ray was also performed, which revealed a widening of the superior mediastinum due to a probable vascular cause. In view of these findings, a thoracic CT scan was requested (Figures 1-3), which demonstrated thickening of the interstitium with ground glass areas, thickening of the subpleural interlobular septa associated with bronchiectasis, large mediastinal vessels with maintained caliber, lusory right subclavian artery that could cause dysphagia lusoria, and atheromatous calcifications of the aorta and coronary territory.

The patient was referred to the cardiothoracic surgery consultation and is awaiting observation.

## Discussion

The severity of dysphagia varies according to its etiology, and diagnosis and determination of its cause are crucial for its therapeutic management [1].

Lusory dysphagia is defined as a swallowing disorder secondary to extrinsic esophageal compression by vascular structures, most commonly being caused by an aberrant right subclavian artery (lusory artery) [3-5]. The most common congenital anatomical variation of the aortic arch is an aberrant subclavian artery, with an estimated prevalence of 0.4% to 2% in the general population and, in most cases, it is asymptomatic, being an incidental finding [1,3,6,7]. In symptomatic adults, the most frequent manifestation is dysphagia.

Patients at any age can develop symptoms, although they usually tend to occur around the end of the fifth decade of life, probably related to changes in the esophagus and blood vessels that occur with advancing age [3,8]. CT-based angiographic studies are the best in diagnosis, considering their availability and non-invasiveness. Esophagogastroduodenoscopy can visualize any mucosal changes or extrinsic compressions [3].

The diagnosis of dysphagia lusoria is complex as the symptoms are frequently non-specific. It is extremely important to diagnose and properly treat dysphagia. This is only feasible through an adequate clinical interview and complementary diagnostic methods that take into account possible differential diagnosis [2,3].

Regarding treatment, adopting a healthy lifestyle and eating behavior education can significantly ameliorate the patient's clinical condition in moderate cases. The surgical approach seems to be controversial, with its indication not having been clearly defined [3,9].

## Conclusions

Although dysphagia is most commonly caused by digestive disorders, lesser common diseases must also be excluded from the diagnostic investigation. These include vascular compression of the esophagus in the mediastinum, namely by an aberrant right subclavian artery. Although dysphagia lusoria is a rare cause of dysphagia in adults, this is a possible cause that should be taken into consideration when making the differential diagnosis of this condition.
